# Impact of an intradialysis virtual-reality-based exercise program on healthcare resources expenditure: a micro-costing analysis

**DOI:** 10.1186/s12882-022-02859-8

**Published:** 2022-06-27

**Authors:** Alicia García-Testal, Francisco José Martínez-Olmos, Jose Antonio Gil-Gómez, Javier Villalón-Coca, Rafael Ortiz-Ramón, Alicia Cana-Poyatos, Rafael García-Maset, Eva Segura-Ortí

**Affiliations:** 1 Nephrology Service, Hospital de Manises, Valencia, Spain; 2 Department of Physiotherapy, Universidad Cardenal Herrera –CEU, CEU Universities, Valencia, Spain; 3 University Institute of Automation and Industrial Technology ai2, Universitat Politècnica de València, València, Spain; 4 Business department, Hospital de Manises, Valencia, Spain

**Keywords:** Exercise, Haemodialysis, Cost

## Abstract

**Background:**

Engagement in exercise by haemodialysis (HD) patients has been shown to generate benefits both in terms of improved functional capacity and in the health-related quality of life. The use of non-immersive virtual reality (VR) games represents a new format for the implementation of intradialysis exercise. Some studies have shown that engaging in exercise for 6 months reduces the consumption of antihypertensive drugs and decreases the time spent admitted to hospital among individuals receiving HD treatments. The objective of this study was to evaluate changes in the consumption of healthcare resources and micro-costing for patients on HD who completed a VR exercise program.

**Materials and methods:**

Design: This study is a secondary analysis of a clinical trial. The participants performed an intradialysis exercise program with non-immersive virtual reality for 3 months. The variables were recorded in two periods: 12 months before and 12 months after the start of the exercise program.

**Results:**

The micro-costing analysis showed a significant decrease in the mean cost, in euros, for the consumption of laboratory tests − 330 (95% CI:[− 533, − 126];*p* = 0.003), outpatient visits − 351 ([− 566, − 135];*p* = 0.003), and radiology tests − 111 ([− 209, − 10];*p* = 0.03) in the 12 months after the implementation of the exercise program relative to the 12 months prior to its start.

**Conclusion:**

The implementation of intradialysis exercise programs decreased the expenditure of some healthcare resources. Future studies could help clarify if longer interventions would have a stronger impact on these cost reductions.

## Introduction

Over the last few years, health spending in many countries has been growing faster than their gross domestic product (GDP). The health expenditure per capita has doubled around the world in less than 20 years. Furthermore, the burden of chronic diseases increases at fast rate in developed countries in the coming decades. On this basis, in-depth knowledge about the cost of chronic disease will be essential [1–3]. End-stage kidney disease (ESKD) has a high incidence and prevalence among patients receiving renal replacement therapy (RRT) and affects more than 500,000 people in Europe, 64% of whom are undergoing treatment with haemodialysis (HD) [4]. Numerous detailed international studies have reported on the excess morbidity and mortality among patients with ESKD with respect to the general population [5, 6].

Patients receiving maintenance HD have reduced physical activity levels compared with their healthy age-matched counterparts and sedentary behaviur is associated with the physical deterioration that results in limitations or restrictions when performing the activities of daily living, gradual deterioration in physical function, and health‐related quality of life, which can increase the number of comorbid conditions they suffer and increase their risk of mortality [7, 8].

All the above generates high levels of consumption of direct and indirect healthcare resources. Some authors from different countries have reported the estimated cost of providing healthcare to patients with chronic kidney disease (CKD) at 2–3 times higher than control patients without CKD [9], and for HD patients, may sometimes be up to 40 times higher. Thus, it seems relevant to search for ways to improve this clinical and economic situation [9–13]. In this sense, the implementation of interventions to increase the physical activity level of this population will probably be important. Many studies have shown the beneficial effects that engaging in different exercise modalities has on patients on HD [14–17].

To facilitate the implementation of exercise engagement in dialysis units, we tested the use of virtual reality (VR) in combination with an intradialysis exercise program in these patients. This technique is a relatively inexpensive and universally applicable tool that can be leveraged to help individuals receiving HD to follow exercise programs by taking advantage of its leisure-like component and game-like nature. VR exercise programs have been used in a diverse range of populations, including those with cerebral palsy,Parkinson’s, or Alzheimer’s disease. In 2017, our research group developed the first reported non-immersive VR program during HD, we compared two groups, conventional exercise vs VR, both intradialysis [18]. This pilot study demonstrated that intradialysis exercise with VR improves physical function and achieves high exercise adherence. Moreover, we have developed a clinical trial with 12 weeks of VR intradialysis intervention that has confirmed these improvements in physical function [19]. So, we verified that intradialysis VR exercise is well tolerated by patients and it positively affects their functional capacity, strength, and health-related quality of life [18–22].

The cost-effectiveness of interventions that promote exercise has been studied in different cohorts and fields and has also shown positive results [23, 24]. However, there is controversy surrounding this topic and more studies are still required to expand the evidence regarding the usefulness of such programs for HD patients [25–27].

Based on the above, our hypothesis in this study was that an exercise program for patients undertaking HD would have a positive impact on the healthcare resource expenditure for these patients. Therefore, our main objective was to evaluate changes in the consumption of healthcare resources and micro-costing among patients on HD who completed an exercise program compared to the same patients in the period prior to this intervention.

## Material and methods

Design: This study is a secondary analysis of a clinical trial (19). This was a randomised, crossover, controlled trial. Eligible participants were randomly allocated either into a group which received the VR intervention for 12 weeks followed by the control treatment for 12 weeks, ‘VR–Control’ (VRC), or vice versa, ‘Control–VR’ (CVR). The variables were recorded in two periods: 12 months before the implementation of the exercise program and 12 months after the start of the program. The participating patients were recruited from the Haemodialysis Unit at the beginning of 2018.

We included patients aged over 18 years who were clinically stable, on maintenance HD treatment for at least 3 months when starting the study, and who had signed their informed consent to participate in the study. We excluded individuals who had suffered a myocardial infarction in the 6 weeks prior, with unstable angina during exercise or at rest, a lower-limb amputation above the knee (without a prosthesis), cerebral vascular disease such as stroke or transient ischemia, skeletal muscle alterations, respiratory diseases that worsened with exercise, people unable to perform functional tests or the planned intervention, and those with a lower-limb vascular access (VA).

The VR exercise intervention was carried out between April and September 2018, lasted 12-weeks, and was implemented and supervised by a physiotherapist with the support of the HD unit nursing staff. Each session during the intervention period comprised a 5-min warm-up followed by the VR exercise session, which lasted a maximum of 30 min, depending on each patient’s individual fitness level, and was completed intradialysis during the first two hours of the HD session.

The exercise program was adapted from a non-immersive VR system game called *Treasure Hunt*, with the aim of making the patient intradialysis exercise session experiences more enjoyable. In this game the players must hunt for objects such as virtual coins while avoiding obstacles like virtual explosives by freely moving their legs, especially by raising their lower limbs up (hip flexion with the knee extended and foot in a neutral position) or left or right (hip abduction and adduction with the knee extended and foot in a neutral position). The difficulty level of the game was graduated according to the patient’s characteristics and the participants could change the leg they used to play the game whenever they became tired.

The hardware we used was a standard desktop computer and monitor screen with a Microsoft Kinect® movement-detection camera. Before starting each session, the physiotherapist defined the VR intervention for each patient by selecting the number of exercise sets and rest periods, and their duration. The game difficulty automatically adapted to match the user’s in-game results: an increased difficulty level meant that more objects appeared for the players to ‘catch’ or ‘avoid’, and they appeared and disappeared at a faster rate. Once the game finished, each patient completed five minutes of gentle stretching. The ideal exercise difficulty level was between 12 and 15 out of 20 on the perceived exertion scale. If the session was too easy (6–11 on the scale) or too hard (16–20) the physiotherapist altered the game settings to make it easier or harder.

The following patient descriptive variables were recorded at baseline: age (in years), sex (male or female), race (Caucasian, Black, or Asian), body mass index (kg/m^2^), diabetes mellitus status (yes/no), current smoking status (yes/no), renal insufficiency aetiology, VA type (arteriovenous fistula [VAF]/central venous catheter [CVC]), HD technique (low-flow HD [LFHD], high-flow HD [HFHD], or online haemodiafiltration (OLFHD), session duration in minutes, blood flow rate (mL/min), dialysate flow rate (mL/min), and the intrasession standardised urea clearance rate (Kt/V) measured using online clearance monitoring (OCM), serum haemoglobin, albumin levels (g/dL) as well as the mean systolic blood pressure and diastolic blood pressure (mmHg), heart rate (in beats per minute [bpm]), for each session.

Comorbidity was quantified using the Charlson Index which assigns weights to different diseases.

Functional capacity was measured using the 6-min walking test (6MWT) in which, when instructed “walk as far as possible for 6 min”, the patient walked up and down a 30-m corridor (which was marked on the ground every two metres) as far as possible over 6 min. The total distance covered (in metres) was recorded.

The result variables were registered during two periods, the first one covered the 12 months prior to the intervention (Period 1) and the second one recorded the 12 months following the start of the intervention (Period 2). Healthcare resource consumption and costs were measured for several variables: the number of episodes and total amount in euros spent on *laboratory tests*, *radiology tests*, *hospital pharmacy and* medical assistance as *Outpatient visits, Emergency healthcare provision*, and *Hospitalization*. To measure consumption, the number of episodes was quantified. To measure costs, micro-costing analysis was used to estimate, in detail, the amount of each resource component used. The information used in this study was derived from different databases linked to each patient’s electronic medical record, as well as from the business resource planning software used for financial management by the healthcare department.

In order to obtain the cost of each of the activities, the information was structured into an in-house cost allocation model based on the ABC (activity-based costing) analytical accounting standard. Briefly, this method divides the production of an organisation into core activities, defines the costs for these activities, and assigns these costs to all the products and services according to the actual consumption of each activity [28, 29]. In this case, the fixed and variable costs considered in this study included supply (medical supplies, pharmacy, energy, etc.), human resources (healthcare and support staff), and financial costs (interests, commissions, and other costs).

### Statistical analysis

The sample size calculation was performed using the main cross-over clinical trial data and was based on detecting changes in physical function as measured by the gait speed test. Thus, we used G*Power software to calculate that a minimum of 16 participants would be required to detect an effect size of 0.459 via ANOVA repeated measures with ‘within-between’ interactions.

Continuous variables were presented as the mean and standard deviation and categorical variables were shown as the relative and absolute frequencies.

Several linear mixed models, one per dependent variable, were adjusted in order to assess the differences between the two periods of time in terms of healthcare costs (in euros) and medical assistance.

All the models also included age, gender, comorbidity, functional capacity, and adherence as covariates (fixed effects) and no interactions were included in the models. To account for the non-independence of observations in the case of repeated measures per patient (variables measured for the same patient in two different periods of time), a random intercept was added to the linear models with the patient used as a random factor. All the statistical analyses were performed with R software (version 3.4.1) mainly by using the lme4 package (version 1.1–17).

## Results

**Fig. 1:**
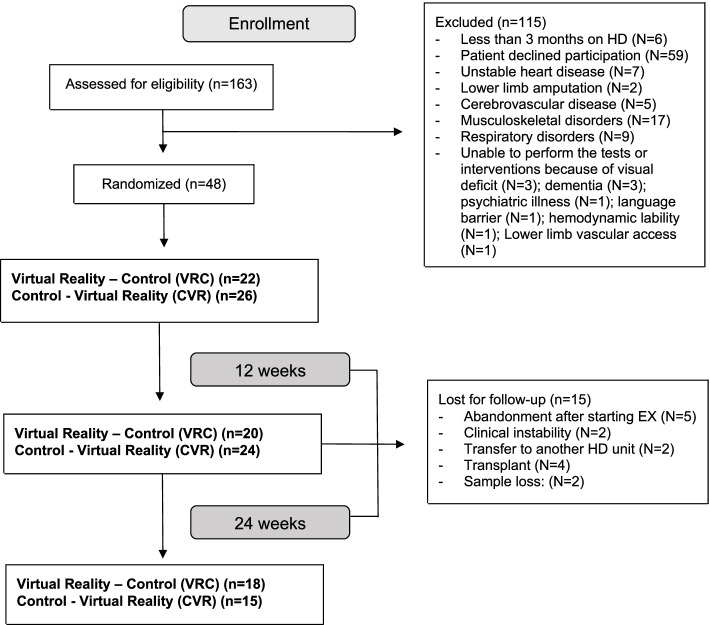
Flow diagram

Fifty-six patients on maintenance HD were included in this cross-clinical trial. Of the 33 patients who had completed the 12-week exercise program, 31 agreed to participate in this sub-study (for a flow diagram of the patient progression though the trial, see Fig. 1). The renal failure aetiology was diabetic nephropathy in 8 patients, glomerulonephritis in 6, vascular renal disease in 5, interstitial nephropathy of urological origin in 1, polycystic renal disease in 1, secondary amyloidosis in 1, renal hypoplasia in 1, and an unknown aetiology in 8 patients. All the participating patients were Caucasian. Functional capacity measured using the 6MWT was 355 (± 120) metres and patient adherence to the exercise sessions was 73% (± 19%). The other patient characteristics are shown in Table 1 and the annual baseline episodes and costs per patient are shown in Table 2.

**Table 1:** Patient baseline characteristics

Participants	*N* = 31
Age (years)	69 (14)
Sex	22 male/9 female
Charlson comorbidity index (points)	6.9 (2.7)
Diabetes mellitus (yes)	14
Cardiovascular disease (yes)	22
Smoking habit (current)	9
Body mass index (kg/m^2^) ± *SD*	27 (6)
Vascular access	26 AVF/5 CVC
HD technique (patients HFHD/LFHD, OLHDF)	20/1/10
HD session duration (minutes)	231 (10)
Blood flow (mL/min)	375 (32)
Dialysate flow (mL/min)	588 (84)
Kt/V	1.5 (0.2)
Systolic blood pressure (mmHg)	138 (19)
Diastolic blood pressure (mmHg)	61 (12)
Heart rate (bpm)	66 (10)
Serum haemoglobin (g/dL)	11.6 (1.4)
Serum albumin (g/dL)	3.8 (0.2)

Results as Mean (*SD*) or Absolute frequencyAVF: arteriovenous fistula, *CVC* central venous catheter, *HFHD* high-flux HD, *LFHD* low-flux HD, *OLHDF* online hemodiafiltration

**Table 2:** Annual baseline episodes and costs per patient

	**Episodes (Number)**	**Cost (Euros)**
Outpatient visits to other specialties	16 (8)	953 (555)
Laboratory tests	23 (7)	1,512 (534)
Radiological tests	3 (3)	2,523 (271)
Hospital pharmacy	2 (3)	1,190 (3803)
Emergency department healthcare provision	0.6 (0.9)	128 (179)
Hospitalisations (hospital overnight stays)	3 (8)	2,396 (5493)

Results as Mean (± *SD*)

The analysis for Period 2 versus Period 1 showed a significant decrease in the number of episodes of outpatient visits to other medical specialties (− 5 ± 1; 95% CI [− 9, − 2]; *p* = 0.004). The changes for episodes of laboratory tests (− 1.4 ± 1.6; 95% CI [− 4.6, 1.8]; *p* = 0.38), radiology tests (− 1.4 ± 0.7; 95% CI [− 2.8, 0.04]; *p* = 0.07), and hospital pharmacy use (− 1.7 ± 0.9; 95% CI [− 3.4, 0.05]; *p* = 0.07) were not statistically significant. The micro-costing analysis showed a significant decrease in costs for the consumption of laboratory tests, outpatient visits, and radiology tests during the Period 2 relative to Period 1 in the 12 months prior to the exercise intervention (Table 3).

**Table 3:** The evolution of the accumulated average cost per patient 12 months from the start of the exercise program

Cost (Euros)	Trend in Period 2	95% confidence interval	*p*
Outpatient visits	− 351	(− 566, − 135)	0.003
Laboratory tests	− 330	(− 533, − 126)	0.003
Radiology tests	− 111	(− 209, − 10)	0.03
Hospital pharmacy	− 579	(− 1,371, 212)	NS
Emergency healthcare provision	42	(− 51, 141)	NS
Hospitalisation	1,219	(− 1,746, 4,184)	NS

*NS* not significant

## Discussion

In this present study we observed that expenditure on healthcare resources significantly decreased among ESKD patients after they participated in an intradialysis VR exercise program. Specifically, there was a reduction in micro-costing in the form of laboratory tests, radiology tests, and outpatient visits after this intervention. We also observed a significant decrease in outpatient visits and their associated costs. Moreover, episodes of laboratory tests, radiology tests, and hospital pharmacy use also decreased, although not to a statistically significant degree. Future studies with longer exercise intervention periods or larger sample sizes may be able to detect decreases in the overall number of episodes and costs per patient in this group.

According to the literature, exercise improves the functional capacity and self-perceived health-related quality of life of ESKD patients receiving HD [14, 22], and this can contribute to a lower healthcare demand by these patients. Previous work also found population decreases in the consumption of other resources after the completion of similar exercise programs. For example, Miller et al. [27] found a 36% reduction in the use of antihypertensive medications with an annual cost savings of $885/patient-year among patients on HD who participated in an aerobic exercise program during dialysis. Parker et al. [26] reported a reduction in hospital stay duration from 7.8 days at baseline to 3.1 days after their 6-month exercise program intervention.

Like this current work, the later study was a retrospective study with patients of similar age, but with a larger sample size and a longer exercise period. They analysed 6 months before and during the implementation of the exercise program. However, in our opinion, it is more informative to compare a full 12-month period in this type of work because different times of the year may have a different incidence of hospital episodes. Of note, our patients had an annual average hospital stay of 3.2 days and we detected no significant changes in this factor after the intervention. As previously stated, future studies could clarify if longer intervention times or larger study cohorts might identify positive results in terms of reduced hospital stays and their associated costs.

The trend towards reduced hospital costs in relation to the implementation of patient exercise programs has also been observed in other populations. For instance, Valero Elizondo et al. [23] studied more than 20,000 participants and estimated that moderate-vigorous physical activity for more than 30 min 5 or more days per week, resulted in a 20% reduction in healthcare expenditure among patients with cardiovascular disease. In systematic reviews, Garret et al. [24] reported that interventions promoting physical activity in primary care appeared to be cost-effective, and estimated the cost-utility at €348 to €86,877 per quality-adjusted life-year gained. Finally, Morton et al. [30] reported a reduction in total hospital costs after an exercise intervention among hospitalised older individuals with acute conditions, compared to their usual care, although they found no effect on the length of hospital stay in these patients.

Interventions to reduce costs are important for every chronic disease, and especially in CKD. Several authors [9–12] have reported global costs in patients with CKD up to 40–60 times higher than those of the general population. ESKD patients have high costs for RRT, but they also have increased costs for medication and hospital stays compared to the general population. Indeed, the annual cost for ESKD patients has been calculated as ranging from €20,000 to €80,000 at different times and in different countries [9, 11, 12]. Thus, exercise interventions that both reduce costs and improve physical function and health-related quality of life among every patient type can be incredibly positive, but especially so among CKD patients.

A limitation was the design of this retrospective observational study, as well as the lack of a control group, were major limitations. In addition, the exercise program intervention we used was only implemented for 3 months, which could have limited the beneficial effects of this program. Finally, it is difficult to generalise conclusions about cost because the respective costs of hospital services in other countries might differ from those in our setting. Thus, longer, controlled interventional studies will be required to fully detect possible decreases in the use of healthcare resources and in their cost in the context of exercise patient interventions.

## Conclusion

The implementation of intradialysis exercise programs results in a decrease in the consumption of some healthcare resources and micro-costs. Future studies could clarify if longer interventions would have a higher impact on the expenditure of these resources.

## Data Availability

The datasets used and analysed during the current study are available from the corresponding author on reasonable request.
